# From Lévy walks to Brownian motion in schools of zebrafish (*Danio rerio*)

**DOI:** 10.1140/epje/s10189-026-00602-x

**Published:** 2026-07-21

**Authors:** Andreza M. da Silva, Francisco C. B. Leal, Pabyton G. Cadena, Viviane M. de Oliveira, Adauto J. F. de Souza, Antonio R. de C. Romaguera, Anderson L. R. Barbosa

**Affiliations:** 1 Departamento de Física, Universidade Federal Rural de Pernambuco, Recife, PE 52171-900 Brazil; 2 Departamento de Morfologia e Fisiologia Animal, Universidade Federal Rural de Pernambuco, Recife, PE 52171-900 Brazil

## Abstract

Population density strongly affects the internal organization and movement dynamics of fish schools. Here, we investigate density-dependent collective motion in zebrafish (*Danio rerio*) schools with group sizes ranging from 2 to 30 individuals. Using trajectories measured relative to the school center of mass, we show that individual motion is superdiffusive at low densities and progressively approaches Brownian-like behavior as density increases. Step-length distributions are well described by truncated power laws: for N≤15, the fitted exponents are compatible with a Lévy-walk regime, whereas larger groups show a transition toward Brownian motion. In parallel, *q*-Gaussian fits of the signed radial displacement increments reveal heavy-tailed, non-Gaussian fluctuations at low densities and a gradual convergence toward Gaussian-like behavior at high densities. These results support a density-driven transition from a polarized, strongly correlated schooling state to a more disordered shoaling state, suggesting that increased density reorganizes local interactions within the group.

## Introduction

The collective behavior of animals constitutes one of the great wonders of the natural world. It represents a fundamental aspect of group life, whose adaptive implications have been widely investigated in the scientific literature [1, 2]. This form of social organization not only reveals the intrinsic characteristics of individuals but also provides valuable insights into the social interactions that emerge within these systems. A central element of this dynamic is leadership, which manifests itself as a regulatory factor in several social groups, such as flocks of birds [3–5], schools of fish [6–9], and herds of horses [10, 11], where hierarchical networks shape collective coordination. Furthermore, studies show that the spatial position occupied by an individual within the collective formation often reflects their social position in the group [12, 13], indicating a relationship between location and hierarchical function. Individual characteristics, such as body size, age, gender, and time spent in the group, also play a relevant role in the emergence and maintenance of these social structures [4, 14].

The dynamics of animal groups have proven to be a promising system for investigating typical phenomena of statistical physics [15–19], including criticality, fractality, and anomalous diffusion [13, 19, 20]. It is no coincidence that most of these models exhibit power laws in their results, since these laws play a central role in describing critical and hierarchical systems such as geometric and statistical fractals [21, 22] as well as in phase transitions [18]. Notable examples of the presence of power laws in collective motion systems include recent investigations on the noise in the trajectories of flocks of starlings (*Sturnus vulgaris* ) [19] and schools of ayu (*Plecoglossus altivelis* ) [23]. In these studies, the mean-square displacement of individuals about the center of mass of the group was analyzed, revealing superdiffusive behavior—that is, individuals move faster than would be expected in a Brownian motion. Furthermore, in the case of ayu schools, it was observed that individual displacements follow patterns compatible with Lévy walks, often associated with efficiency in search strategies [24, 25].

The dynamics of zebrafish (*Danio rerio*) schools have been examined using techniques that analyze collective motion and identify power-law patterns in their behavior. A recent study applied the Multifractal Detrended Fluctuation Analysis (MF-DFA) technique to investigate the polarization time series of schools at varying fish densities [16]. The findings revealed that, at low densities, the polarization velocity of schools displays non-Gaussian behavior with long-range correlations. Conversely, at high densities, the behavior shifts to Gaussian and uncorrelated. This transition was interpreted as an out-of-equilibrium phase transition, from a schooling to a shoaling phase, highlighting the influence of density on fish synchronization [15, 16, 26]. In the context of the schooling-to-shoaling phase transition, it has been shown that stress induced by increased density causes the shoal to fragment into smaller domains [15]. Within these domains, individuals exhibit strong correlations, while interactions between domains are significantly weaker. The observed spatial modularity appears to facilitate the transition from polarized to unpolarized collective states, suggesting an adaptive response to increased density and the reorganization of social interactions within the group. One of the explanations for such rich behavior is provided in [27], where the authors demonstrate the existence of multiple time scales, ranging from 1 to 5, as a function of fish density.

Stress caused by the confinement of fish in small spaces plays a vital role in preclinical studies, particularly because confinement has been shown to induce marked physiological and behavioral responses in teleosts, such as elevated cortisol levels and altered locomotor activity [28]. Moreover, zebrafish are widely used as models for neurodegenerative diseases such as Parkinson’s disease, where neurotoxins like MPTP reproduce dopaminergic and locomotor deficits [29]. Investigating how confinement stress affects the collective behavior of healthy fish schools can therefore provide a baseline for interpreting disease-related behavioral alterations observed in such models more effectively. A promising approach involves analyzing how noise in individual trajectories evolves with increasing population density, which parallels intensifying confinement stress. Consequently, it is also possible to investigate the relationship between this behavioral phase transition and changes in the stochastic pattern of trajectories.

In this work, we investigate the dynamics of individual fish trajectories about the center of mass of zebrafish (*Danio rerio*) schools, varying the population density of the groups. Through the analysis of the mean-square displacement, we verify that, in low-density schools, the trajectories of individuals around the center of mass exhibit a superdiffusive behavior. As the density increases, we observe a gradual transition to a regime that approaches normal diffusion, in which individual movements become similar to those of Brownian walkers, although superdiffusion characteristics persist.

**Fig. 1:**
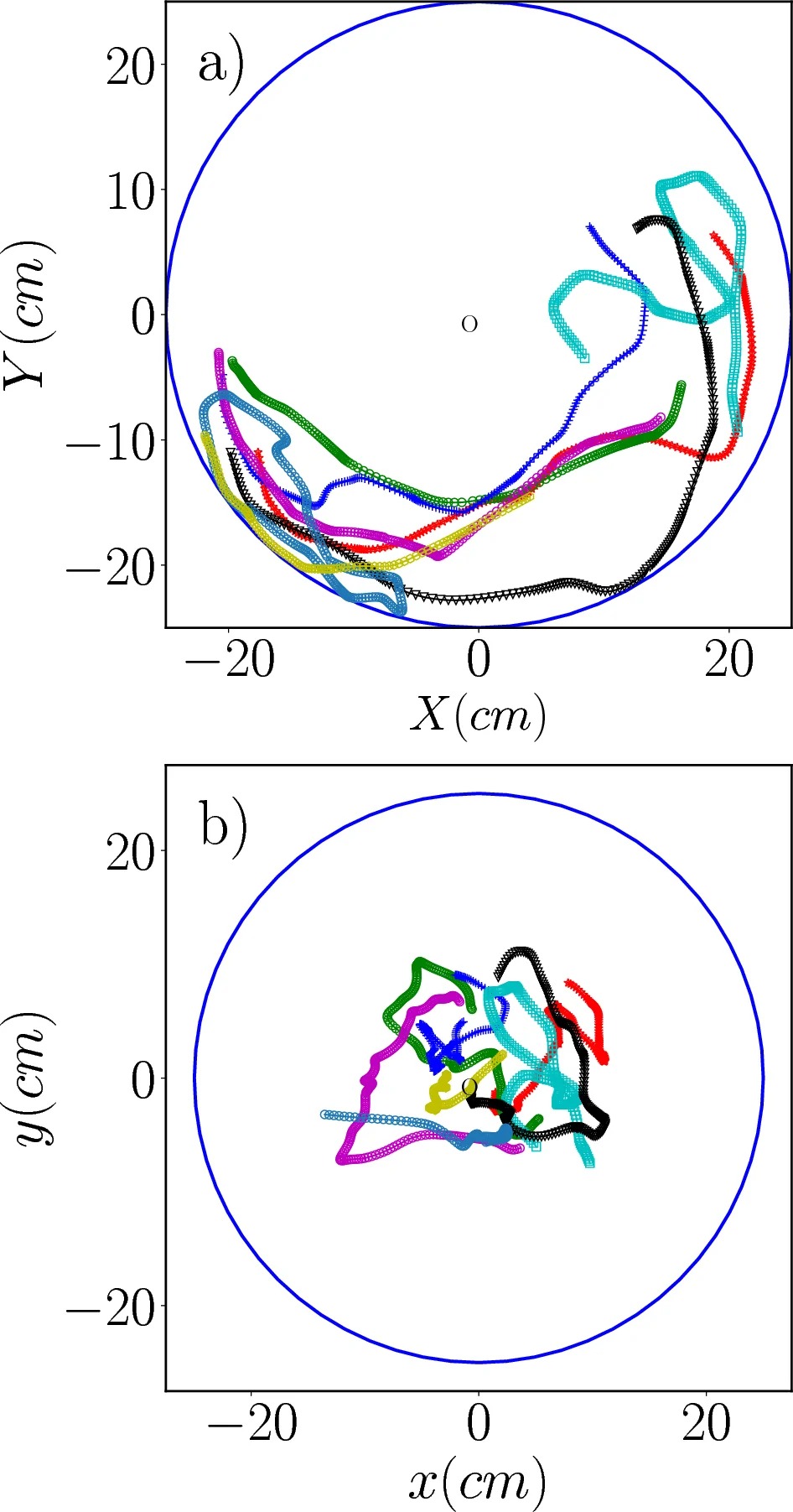
**a** Experimental trajectories of 8 adult zebrafish in a circular tank with a radius of 25 cm, recorded over a 60 s time window. **b** The corresponding trajectories in the center-of-mass reference frame

Furthermore, we investigated the presence of Lévy-walk patterns in individual trajectories relative to the group center of mass. We identified typical Lévy-walk signatures for low-density schools, corroborating the results obtained by the mean-square displacement analysis. For higher fish density, we observed a mixed behavior, in which Brownian-like and Lévy-walk patterns coexist. We can see a change in behavior through the graphs of the trajectories in two different reference frames. Figure 1a shows the trajectories of a few animals in the reference frame of the circular tank. In contrast, in Fig. 1b, the same trajectories are shown using the group center of mass as a reference frame. In this second case, the trajectories exhibit numerous small displacements interspersed with rare large jumps—a characteristic Lévy-walk pattern.

To statistically characterize these dynamics, we used q-Gaussian distributions [30] to model the trajectory noise relative to the center of mass. Our results indicated that, in low-density schools, the distributions present heavy tails, consistent with Lévy-walk dynamics. However, with increasing density, the distributions gradually converge to Gaussian behavior, evidencing a stochastic transition that reflects the change in individual locomotor strategies. This result reinforces previous observations on the influence of density on the organization of group dynamics [15–17, 26].

## Experimental setup

In this study, experiments were conducted with groups of fish of different sizes, composed of 2, 3, 5, 8, 10, 13, 15, 18, 20, 25, and 30 individuals. We used a total of 115 adult fish, all approximately one and a half years old, with an average length of 3.0 cm. Observations took place in the morning, between 8:00 AM and 12:00 PM (Brazil time) from March to September of 2022. The experiment was carried out in a circular tank with a diameter of 50 cm and a water depth of 5 cm, maintained at a constant temperature of $24.5^{\circ }$C. During each trial, a specific number of fish were randomly selected and placed in the tank, with their physical characteristics carefully controlled to ensure the accuracy of the results.

**Fig. 2:**
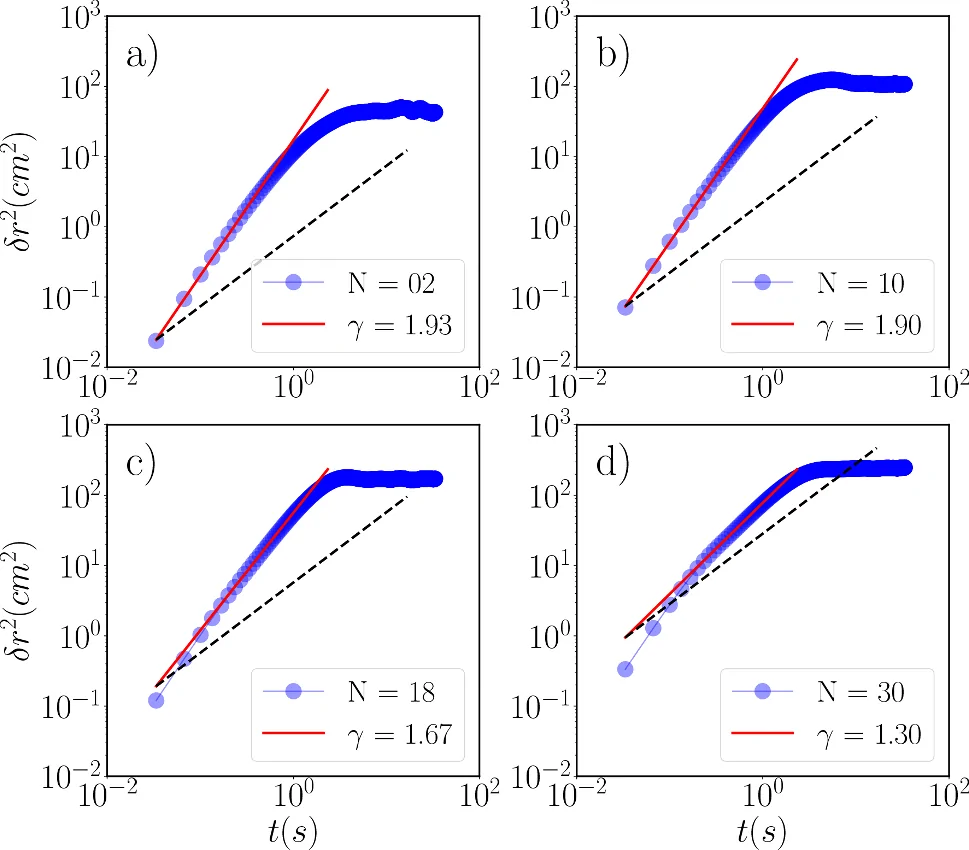
Mean-square displacement relative to the center of mass for schools with **a** 2 individuals, **b** 10 individuals, **c** 18 individuals, and **d** 30 individuals. The black dashed line represents the theoretical Brownian diffusion with exponent γ=1, while the red solid line shows the measured group dynamics. The corresponding exponent γ is displayed in the inset

The experiment was conducted in a tank illuminated by diffuse LED light, with white walls surrounding it to maintain uniform lighting and minimize external interference. Filming was done with a full-frame DSLR camera equipped with a 50-mm lens, positioned 1.46 m above the tank. The camera captured black-and-white images at a resolution of 1920×1080 pixels and a frame rate of 30 frames per second. Before recording, the fish were left in the tank for 10 minutes to acclimate to the environment. For data collection, the idTracker software was used to digitize the footage and obtain the positions of the fish over time. The analysis considered a total recording time of 6.5 minutes per trial. The fish trajectories were smoothed using the Tikhonov regularization method [31] for precise data fitting. Five measurements were taken for each group size *N*, using different sets of fish for each trial to ensure statistical independence. All experimental results presented in the following sections, including displacement dynamics and noise distributions, represent the average over these five independent realizations. Uncertainties and error bars correspond to the standard deviation calculated across these trials.

## Analysis and discussion

### Mean-square displacement

We investigated the diffusive behavior of fish in their schools. The mean-square displacement (MSD) is a fundamental measure quantifying a particle’s position deviation from a reference point over time. This metric is widely used to characterize the spatial extent of random motion and allows for determining whether a particle spreads slowly or rapidly, influenced by the system’s internal correlations. In this study, we calculated the displacement of each individual within the school using the mean-square displacement relative to the center of mass of the group as a function of time (t) [19, 23]:

1 $$\begin{aligned}  &   \delta r^2(t) = \frac{1}{T - t} \frac{1}{N} \sum _{t_{0} = 0}^{T - t - \delta _{0}} \sum _{i = 1}^{N} \left| \textbf{r}_{i}(t_{0} + t) - \textbf{r}_{i}(t_{0}) \right| ^2,\nonumber \\ \end{aligned}$$

where $\delta _{0}$ is the experimental acquisition time equal to 1/30 s.

In this context, $\textbf{R}_{i}(t)$ denotes the position of fish *i* at a given time *t* relative to the tank frame, while $\textbf{R}_{CM}(t)$ represents the position of the center of mass of the school at the same time. Consequently, the relative position of each fish concerning the center of mass, $\textbf{r}_{i}(t) = \textbf{R}_{i}(t) - \textbf{R}_{CM}(t)$, was determined. To compute MSD, we average over all *N* individuals and over times for which the interval [0, *t*] lies entirely within the observation window, i.e., $t_0 \in [0, T - \delta _0]$. This time-averaging procedure samples all statistically valid displacement increments of duration *t*, ensuring robust estimation of the MSD as a function of the lag time *t*.

Simple diffusion in two dimensions is characterized by the mean-square displacement $\delta r^{2}(t) = 4Dt$, where $\delta r^{2}(t)$ denotes the MSD in the asymptotic long-time limit and *D* is the diffusion coefficient [32]. This type of motion, known as Brownian motion, is associated with Gaussian displacement distributions, as predicted by the central limit theorem [33]. When diffusion deviates from this linear scaling at sufficiently long times, it is classified as anomalous diffusion [34]. In general, the anomaly is quantified by the exponent γ, defined through

2 $$\begin{aligned}  &   \delta r^{2}(t) = 4D t_0~\left( \frac{t}{t_0}\right) ^{\gamma }. \end{aligned}$$

The inclusion of $t_0$ ensures dimensional consistency by rescaling time into dimensionless units of sampling frequency, while the prefactor $4D t_0$ preserves the correct units of $\delta r^2$ and the diffusion coefficient, even for non-integer exponent. For γ=1, this expression reduces to the standard Brownian diffusion law. In the case of superdiffusion (γ>1), the MSD grows superlinearly in time, indicating enhanced transport, whereas subdiffusion (γ<1) leads to sublinear scaling, characterizing a dispersive transport regime [32, 35]. Figure 2 shows the time dependence of $\delta r^{2}(t)$ for fish schools of size N=2,10,18, and 30. For all school sizes, the data follow a power-law scaling $\delta r^{2}(t) \sim t^{\gamma }$ with γ>1 and fit to Eq. (2) confirming this superdiffusive behavior. After a critical time interval, the curves reach a plateau, reflecting the restriction of dynamics imposed by spatial confinement.

**Fig. 3:**
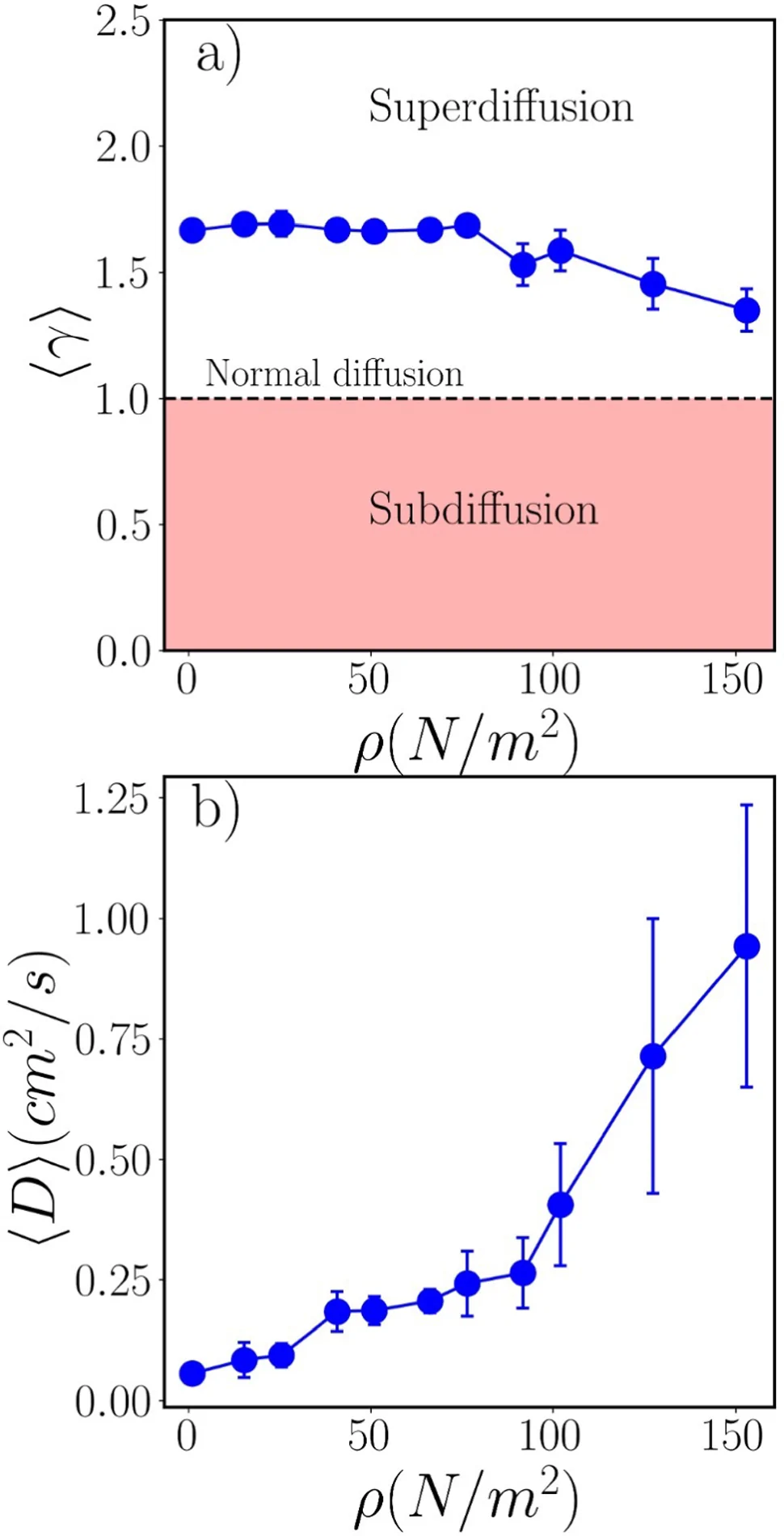
**a** Diffusion exponent as a function of fish density. **b** Diffusion coefficient as a function of fish density

The power-law fit was performed on a specific region of the curve $\delta r^{2} (t) \times t$. As can be seen in Fig. 2, there is a plateau in the region of large values of *t*, caused by the finite size of the experimental circular tank, as discussed previously . Therefore, we excluded from the power-law fit the region where $t > t_{max}$, where $t_{max}$ is the point at which the curve transitions from the region with power-law behavior to the plateau. We also excluded the region where *t* is close to zero since this part of the curve is strongly affected by statistical noise. The fit starts from a minimum value $t_{min}$, defined as the threshold above which the tail of the curve starts to be analyzed, and ends at $t_{max}$. The fit was therefore performed only in the interval $t_{\min }<t<t_{\max }$, where the MSD curves display power-law scaling.

To determine these cutoff points objectively, we performed a spectral convergence analysis. The lower threshold, $t_{min}$, was defined by the frequency cutoff $f_c$, identified as the point where the power spectral density (PSD) of the relative trajectories converges to the instrumental white noise floor (see Appendix A). This approach ensures that the MSD analysis is performed strictly within the signal regime, effectively filtering out high-frequency artifacts from the tracking system. We established a global value of $t_{min} = 1/f_c \approx 0.07$ s for all experimental conditions, as the noise floor was found to be consistent across different group sizes.

Figure 3a shows the values of the exponent γ as a function of the fish density ρ, defined as the ratio between the total number of individuals *N* and the tank area, $A = \pi \times (25~\textrm{cm})^2$.

It can also be seen that, up to $\rho = 13/A = 66.21~\textrm{m}^{-2}$, all data points belong to a plateau with $\gamma \approx 1.7$. On the other hand, from $\rho = 15/A = 76.39~\textrm{m}^{-2}$ onward, γ decreases with increasing density, although the motion remains superdiffusive, since γ>1.

The diffusion coefficient *D* increases monotonically with ρ, as shown in Fig. 3b.

Previous studies report a behavioral phase transition in this same density range, with a critical point at $\rho _c$ [16, 17, 26]. This transition from a polarized state (schooling) to an aggregated but depolarized state (shoaling) is characterized in Ref. [16] using the same dataset analyzed here. In that work, multifractal analysis (MF-DFA) revealed that for $\rho < \rho _c$, the system exhibits long-range memory and high alignment, whereas for $\rho > \rho _c$, the dynamics become monofractal and weakly correlated. This contrast explains why the exponent γ in Fig. 3a approaches 2 for low densities and decays after the critical threshold. Here, we extend these findings by focusing on the stochastic dynamics of the individual trajectories and the resulting noise statistics.

**Table 1:** Values of the diffusion exponent γ, diffusion coefficient *D*, Lévy-walk exponent μ, and *q*-Gaussian parameter *q* for each group size *N*

*N*	2	3	5	8	10	13	15	18	20	25	30
$\rho ~(\textrm{m}^{-2})$	10.19	15.28	25.46	40.74	50.93	66.21	76.39	91.67	101.86	127.32	152.79
$\langle \gamma \rangle$	1.66±0.03	1.70±0.02	1.70±0.05	1.67±0.02	1.66±0.03	1.67±0.01	1.69±0.04	1.53±0.08	1.59±0.08	1.45±0.10	1.35±0.01
$\langle D \rangle$	0.06±0.01	0.08±0.04	0.10±0.02	0.18±0.04	0.19±0.03	0.21±0.02	0.24±0.07	0.30±0.07	0.41±0.13	0.71±0.30	0.94±0.30
$\langle \mu \rangle$	1.83±0.37	1.69±0.48	1.84±0.18	1.89±0.43	2.10±0.11	1.97±0.12	2.22±0.10	3.79±0.24	3.55±0.38	3.41±1.66	4.95±1.74
$\langle q \rangle$	1.72±0.17	1.56±0.06	1.64±0.17	1.73±0.20	1.55±0.12	1.50±0.10	1.58±0.13	1.53±0.10	1.38±0.11	1.31±0.11	1.25±0.08

**Fig. 4:**
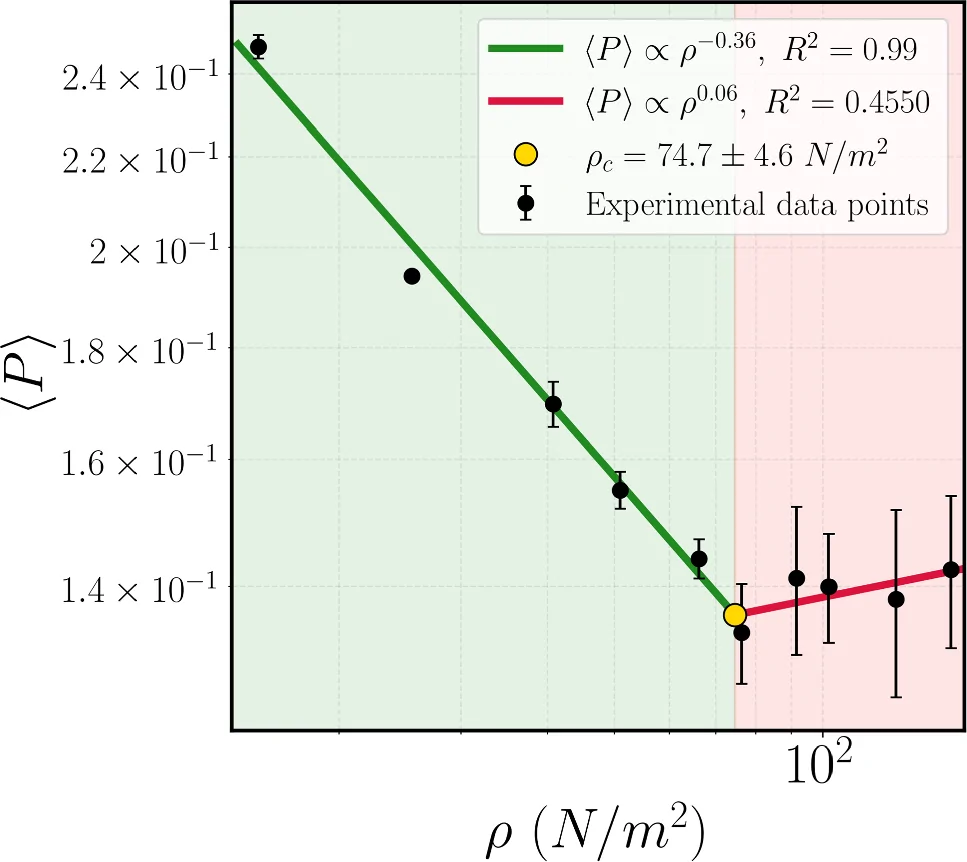
Average polarization $\langle P \rangle$ as a function of fish density ρ in the center-of-mass reference frame. The double power-law behavior reveals a transition at $\rho _c \approx 74.7$ m$^{-2}$. The green line represents the fit for the polarized regime ($\rho < \rho _c$), and the red line shows the plateau for the depolarized regime ($\rho > \rho _c$)

To complement these previous findings, we analyzed the polarization *P*(*t*), defined as the norm of the average unit velocity vectors of the individuals relative to the school’s center of mass: $P(t) = \frac{1}{N} \left| \sum _{i=1}^{N} \vec {v}_{i,cm}(t)/|\vec {v}_{i,cm}(t)| \right|$. Figure 4 shows the time-averaged polarization $\langle P \rangle$ as a function of the school density ρ. We observe a clear behavioral transition at a critical density $\rho _c = 74.7 \pm 4.6~\textrm{m}^{-2}$, consistent with previous findings obtained in a fixed reference frame [16]. This critical value lies between the densities corresponding to N=13 and N=15 individuals.

For low densities ($\rho < \rho _c$), the polarization follows a power-law decay $\langle P \rangle \propto \rho ^{-0.36}$ ($R^2 = 0.99$), indicating that as the number of fish increases in this regime, the relative alignment decreases systematically. Conversely, for high densities ($\rho > \rho _c$), the polarization remains nearly constant and low ($\langle P \rangle \propto \rho ^{0.06}$), characteristic of a depolarized shoaling phase. This transition point $\rho _c$ acts as a threshold that influences several macroscopic quantities of the system, providing the physical context for the changes observed in individual trajectory statistics discussed in the following sections.

### Lévy walks

A Lévy walk is a stochastic process in which, at each discrete step *j*, the active particle performs a displacement of length $l_j$, drawn from a probability distribution function $P(l_j)$. This distribution has a heavy tail that decays as a power law for large $l_j$:

$$\begin{aligned} P(l_j) \sim l_j^{-\mu }, \quad \left\{ \begin{array}{ll} \mu = 1 &  \text {linear trajectories}, \\ 1< \mu < 3 &  \text {L}\acute{\textrm{e}}\text {vy walk}, \\ \mu = 3 &  \text {Brownian motion}. \end{array} \right. \end{aligned}$$

where the exponent μ controls the relative weight of long jumps [25].

The exponent μ plays a fundamental role in characterizing the nature of the movements. Values of μ close to 1 indicate more linear trajectories, while values close to 3 describe a Brownian motion.

**Fig. 5:**
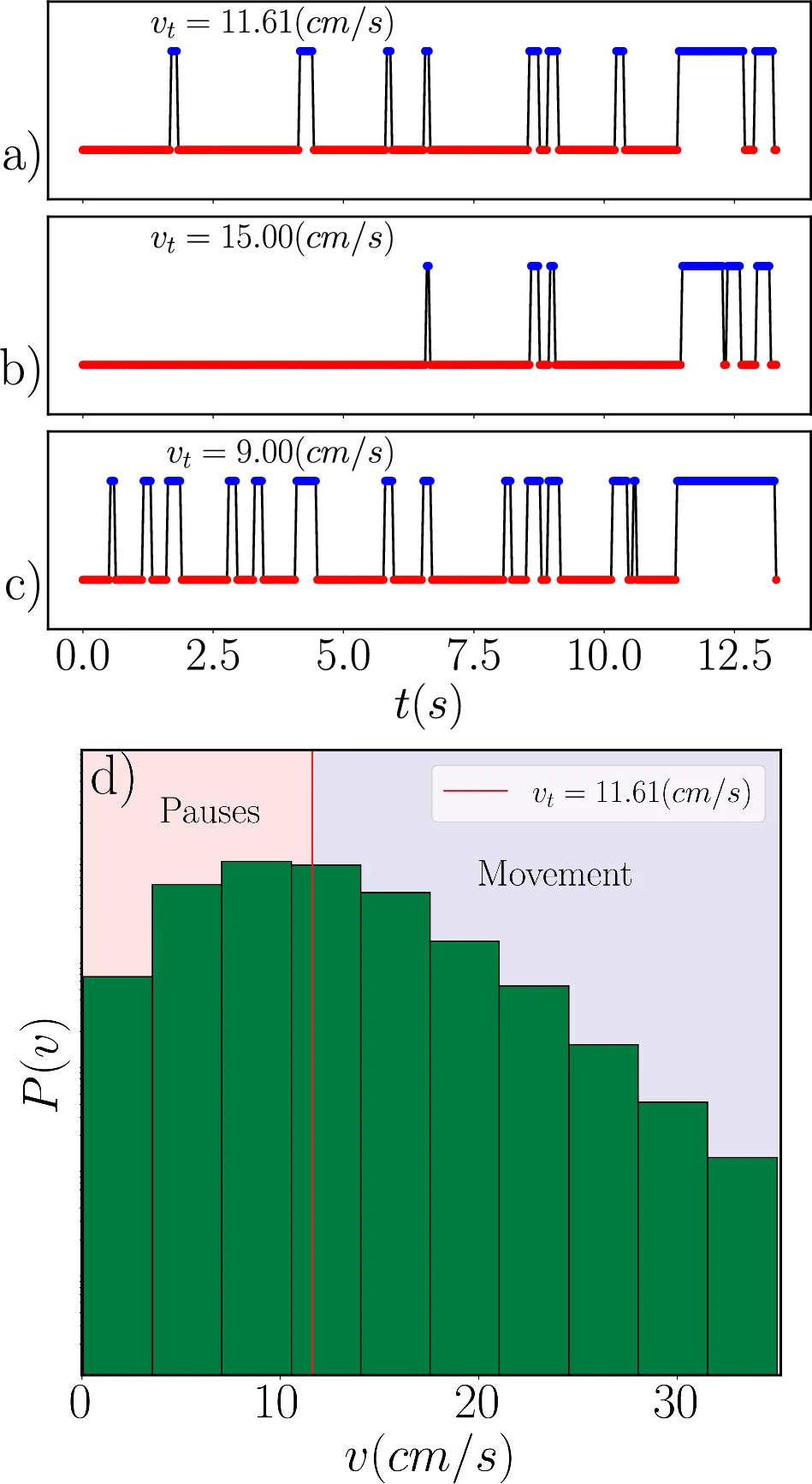
Examples of identification of pause (red) and movement (blue) clusters for different velocity thresholds: **a** $v_t = 11.61~\text {cm/s}$ (this value determined automatically by Otsu’s method), **b** $v_t = 15.00~\text {cm/s}$, and **c** $v_t = 9.00~\text {cm/s}$. **d** Distribution of velocities *P*(*v*) for a fish in a school of 10 individuals, with the threshold $v_t = 11.61~\text {cm/s}$ indicated by the vertical line

We analyze the distribution of the fish displacement lengths relative to the school’s center of mass and evaluate whether these displacements, on a logarithmic scale, follow a power-law distribution. Initially, we applied a protocol to identify pause points: if the average velocity between two consecutive positions of fish *i* relative to the center of mass, given by $|\textbf{r}_{i}(t) - \textbf{r}_{i}(t - dt)| / dt$, is less than a threshold $v_{t}$, we classified the position $\textbf{r}_{i}(t)$ as a pause point. However, if $v_{t} < |\textbf{r}_{i}(t) - \textbf{r}_{i}(t - dt)| / dt$, we classified the position $\textbf{r}_{i}(t)$ as a movement point. In this way, the time series is binarized, assigning 0 to pause points and 1 to movement points. We then identify the clusters corresponding to these categories, as illustrated in Fig. 5a, b, and c. We define the length of a step, ℓ, as the displacement of the fish between the first point of a pause cluster and the last point of the subsequent movement cluster, i.e., a pair of *pause-movement* states.

To determine the optimal value of $v_{t}$ for each school size, we employed an adaptation of Otsu’s thresholding method [36]. This technique segments grayscale images by separating foreground from background, based on differences in pixel intensity. The method assumes that the object exhibits a texture or tone that contrasts with the surrounding environment.

In the present work, we replaced the gray-level analysis of each pixel with the analysis of the speed magnitudes of each fish in the school, seeking to identify the time intervals in which the fish movement dynamics are paused in contrast to the intervals of active movement. In the time series shown in Fig. 5b and c, the binarized dynamics were obtained using arbitrarily chosen speed thresholds $v_{t}$, whereas in Fig. 5a, the threshold $v_{t}$ was determined using the adapted Otsu method.

The method uses a histogram, as shown in Fig. 5(d), to divide the velocity frequencies into two groups: (i) the set $C_{0}$, which includes velocities below a threshold value $v_{t}$, and (ii) the set $C_{1}$, which includes velocities above that threshold. In essence, the procedure determines an optimal value for $v_{t}$ by satisfying the condition of minimizing the within-class variance ($\sigma _{W}^2$), given by:

3 $$\begin{aligned}  &   \sigma _{W}^2 = w_{0} \sigma _{0}^2 + w_{1} \sigma _{1}^2, \end{aligned}$$

where $w_{0}$ and $w_{1}$ are the probabilities of belonging to classes $C_{0}$ and $C_{1}$, while $\sigma _{0}^2$ and $\sigma _{1}^2$ represent the velocity variances for classes $C_{0}$ and $C_{1}$, respectively. See Appendix B for more details.

The Otsu method does not strictly depend on the bimodality of the distribution. Although frequently associated with bimodal histograms in image processing, its mathematical foundation—as established in the seminal 1979 work [36]—is based on the maximization of the between-class variance ($\sigma _b^2$), which is mathematically equivalent to minimizing $\sigma _W^2$. As the author emphasizes, the optimality criterion is derived from the global integration of the histogram’s statistical properties rather than the detection of local features, such as peaks or valleys.

School step lengths follow a truncated power-law distribution characterized by the exponent μ. To ensure statistical rigor in the fit, the lower bound of the scaling regime ($\ell _{min}$) was determined using the Kolmogorov–Smirnov (KS) statistic [37]. Furthermore, the superiority of the truncated power-law model over pure power-law and exponential alternatives was confirmed through a log-likelihood ratio test (p<0.05). The results of this comparison for a representative group size (N=10) are summarized in Table 2.

**Fig. 6:**
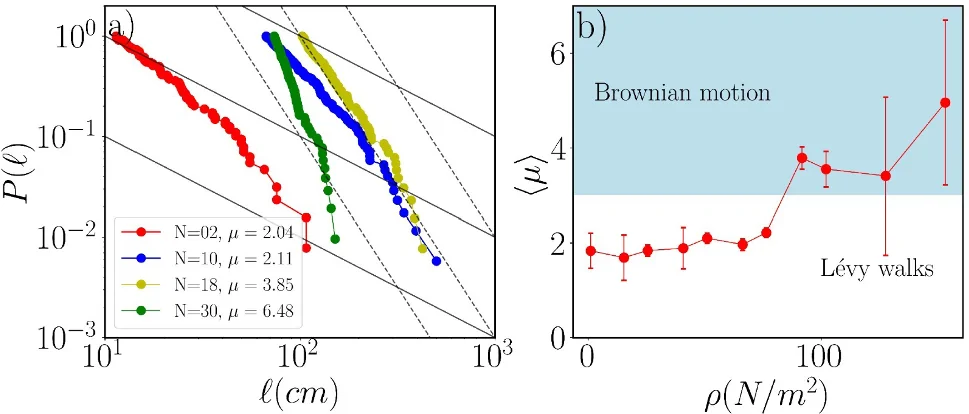
**a** Cumulative distributions of step size for four schools of fish. Schools with 2, 10, 15, and 30 individuals. The solid black line represents the curve with μ=1 and the dashed black line the curve with μ=3. **b** Power-law exponent represented in the power-law relation applied to the step size distribution as a function of fish density

In Fig. 6a, we present the cumulative distribution of step length for four fish schools, while Fig. 6b presents the exponents μ obtained for all group sizes averaged over the five independent realizations. For schools with $\rho < \rho _{c}$, the values of μ vary systematically in the interval $1 < \mu \le 3$, indicating that the fish exhibit Lévy-walk dynamics in the center-of-mass frame. In contrast, for $\rho > \rho _{c}$, we find the most likely value of μ>3. These findings align with previous mean-square displacement analysis, which showed that fish exhibit Brownian-like motion for large groups, while for $\rho \le \rho _{c}$, their motion exhibits superdiffusive behavior.

**Table 2:** Statistical parameters and model comparison for the step-length distributions of N=10 zebrafish across five independent realizations. *R* denotes the log-likelihood ratio between the truncated power-law and exponential models, and *p* is the associated *p*-value. Positive values of *R* indicate preference for the truncated power-law model, while negative values favor the exponential model. Small *p*-values (p<0.05) indicate that the difference between models is statistically significant, rejecting the null hypothesis that both models are equally likely

N = 10	1	2	3	4	5
Sample size (*n*)	713	602	568	594	922
$\ell _{min}$ (cm)	20.98	20.39	21.44	21.09	13.50
**Exponent** μ	2.11	1.86	2.04	2.39	1.89
*R*	**20.0**	**11.5**	**11.0**	**28.2**	**31.8**
*p***-value**	$4\times 10^{-3}$	$2\times 10^{-2}$	$1\times 10^{-2}$	$7\times 10^{-4}$	$4\times 10^{-3}$

We clarify that defining the step length based on pause-movement-pause intervals is grounded in the intermittent nature of fish swimming (burst-and-coast), a methodology well-established in the literature for similar species [23]. Unlike idealized theoretical models that assume perfectly straight ballistic segments between reorientation events, our approach acknowledges that animal movement is continuous and inherently noisy. By adopting the net displacement between successive quiescent states, we filter out small-scale fluctuations and intra-step course corrections that do not contribute to the individual’s effective transport in space. In the center-of-mass reference frame, this displacement represents the fundamental unit of spatial redistribution and local social interaction, capturing the essence of heavy-tailed statistics without the interference of high-frequency instrumental noise.

To ensure that the observed Lévy-like behavior is not an artifact of the data processing stage, we tested the sensitivity of the scaling exponent μ to the choice of the velocity threshold $v_t$. The segmentation of trajectories into discrete steps depends on this parameter, which we objectively determined using the Otsu method ($v_{to}$). The method identifies the optimal threshold ($v_{to}$) by maximizing the variance between the two modes of the relative velocity distribution (see Fig. 15 and Appendix B). To ensure that the observed Lévy-like behavior is not an artifact of this specific choice, we performed a sweep of $v_t$ within a $\pm 10\%$ neighborhood of $v_{to}$ for a representative school size (N=8).

**Fig. 7:**
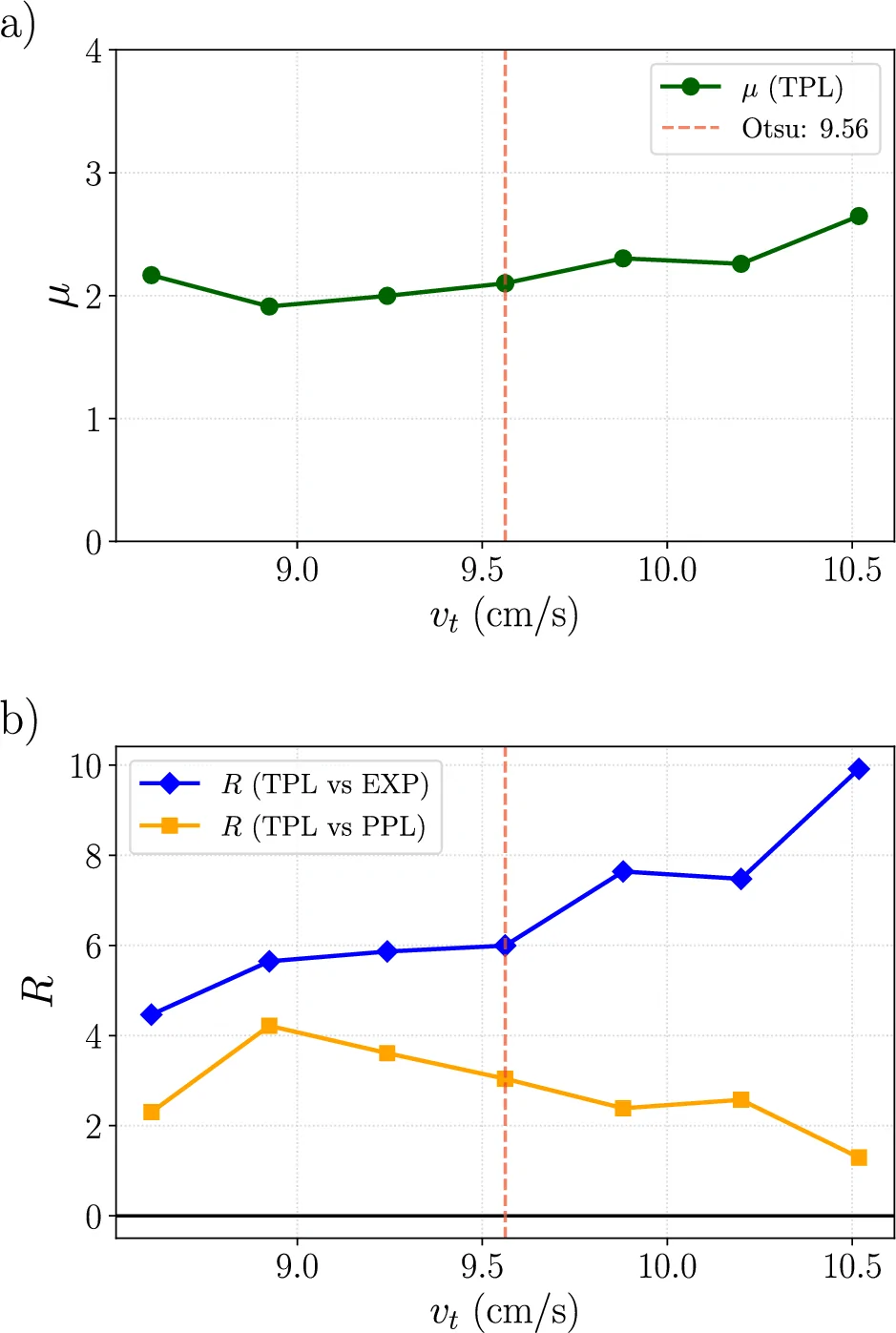
Robustness analysis of the Lévy-walk parameters as a function of the segmentation threshold $v_t$. **a** Stability of the truncated power-law exponent μ, showing consistent values within the superdiffusive range. **b** Log-likelihood ratio *R* for the truncated power law (TPL) compared to exponential (blue) and pure power law (orange) distributions. Positive values of *R* across the $\pm 10\%$ neighborhood of $v_{to}$ confirm that the TPL is the most robust model to describe the step-length statistics

As shown in Table 3 and Fig. 7a, the scaling exponent μ remains remarkably stable across the entire range, with values consistently within the superdiffusive regime ($1 < \mu \le 3$). Furthermore, the log-likelihood ratio tests (*R*) presented in Fig. 7b, along with their respective *p*-values, confirm that the truncated power law (TPL) remains the most statistically supported model even under threshold variation. This consistent superiority of the TPL over pure power-law (PPL) and exponential (EXP) alternatives (R>0) strengthens the argument that the heavy-tailed step-length distribution is an intrinsic property of the collective motion rather than a consequence of the segmentation parameters.

**Table 3:** Statistical robustness of the fit parameters and model comparison for velocity thresholds in the vicinity of the Otsu limit ($v_{to}$)

$v_t (cm/s)$	μ(TPL)	TPL vs. EXP		TPL vs. PPL		KS stat.
		*R*	*p*	*R*	*p*	
8.606	2.17	4.46	0.091	2.30	0.032	0.0526
8.925	1.91	5.65	0.065	4.22	0.004	0.0487
9.243	2.00	5.86	0.049	3.61	0.007	0.0413
9.562	2.10	5.99	0.048	3.04	0.014	0.0425
9.881	2.30	7.64	0.022	2.39	0.029	0.0402
10.199	2.26	7.48	0.025	2.57	0.023	0.0415
10.518	2.65	9.92	0.008	1.29	0.108	0.0347

**Fig. 8:**
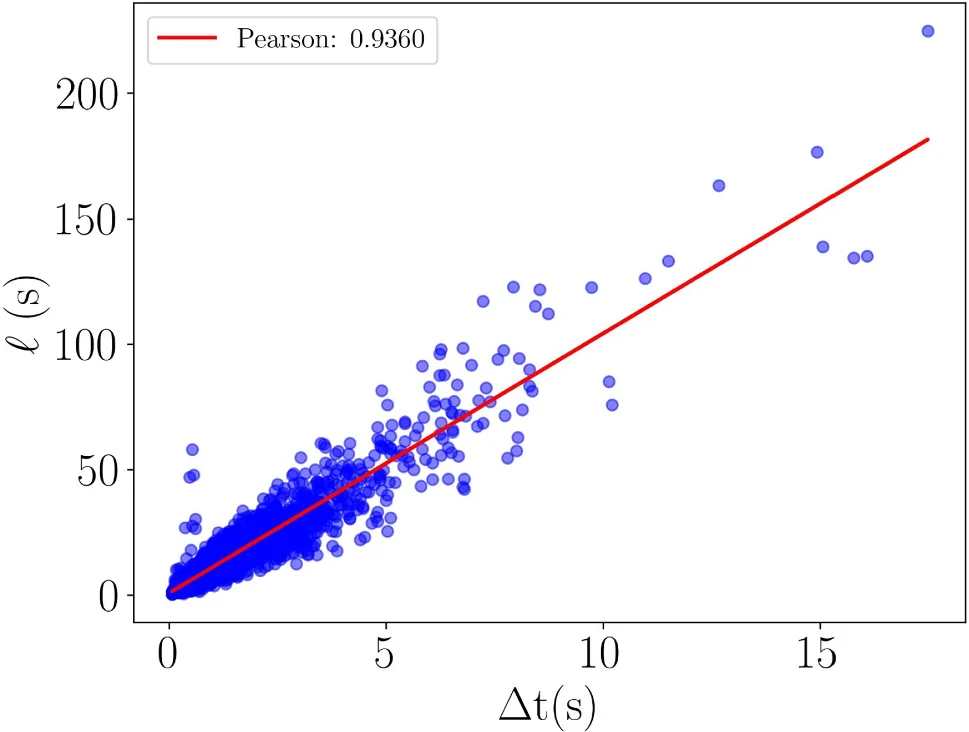
Joint statistics of step lengths (ℓ) and durations (Δt) for a group of N=10 individuals. The solid red line represents a linear fit with a Pearson correlation coefficient of r≈0.94. This strong linear coupling confirms that displacements occur at a characteristic velocity, identifying the dynamics as Lévy-walk dynamics rather than an idealized Lévy flight with instantaneous jumps

To further validate the choice of the Lévy-walk model over idealized Lévy flights, we examined the coupling between step lengths (ℓ) and their respective durations (Δt). In biological systems, movement occurs at finite velocities, whereas idealized Lévy flights assume instantaneous jumps where distance and time are uncoupled ($\Delta t \rightarrow 0$ or independent of ℓ). For a representative school size (N=10), we observed a remarkably strong linear correlation between these variables, as shown in Fig. 8 (Pearson coefficient r≈0.94).

This tight coupling indicates that individual displacements follow a characteristic and approximately constant propagation speed, such that $\ell \sim \Delta t$. The high value of *r* implies that the ratio $v = \ell / \Delta t$ remains stable across different scales of displacement. This linear relationship is a hallmark of Lévy walks and justifies our transition to this terminology throughout the manuscript, as it accurately reflects the continuous nature of zebrafish trajectories moving at finite velocities.

**Fig. 9:**
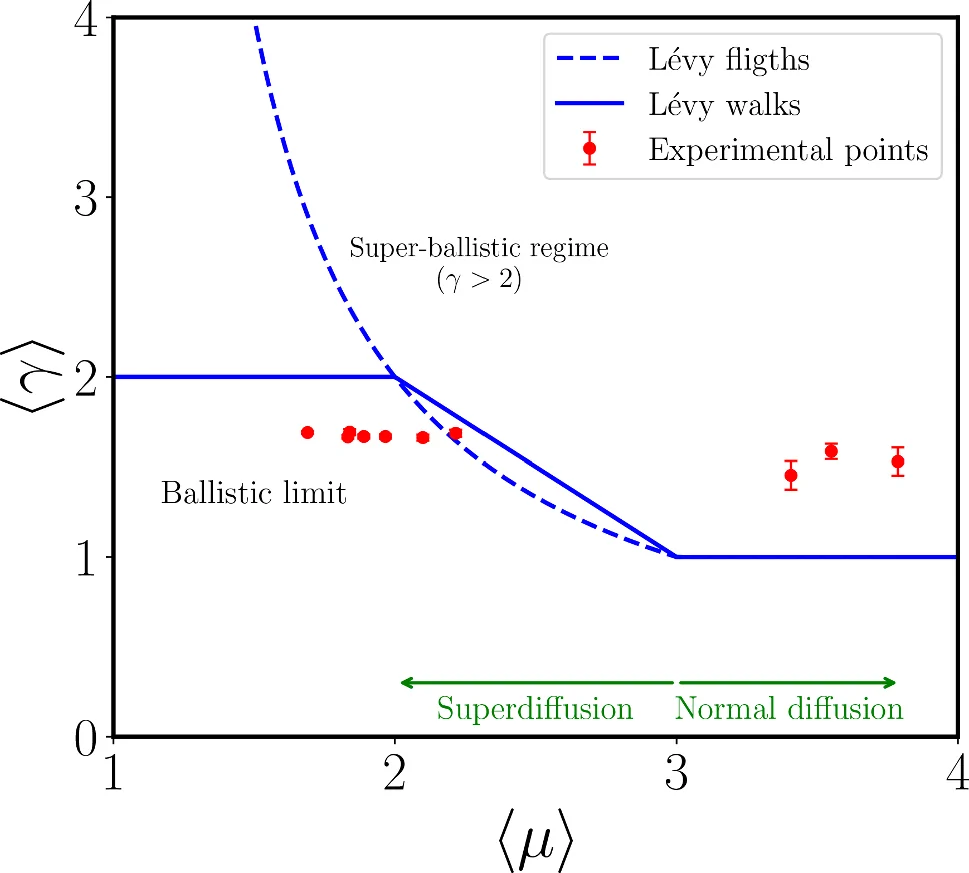
Cross-check of theoretical scaling relations between the MSD exponent (γ) and the step-length tail index (μ). The solid blue line represents the Lévy-walk prediction ($\gamma = 4 - \mu$), while the dashed blue line indicates the Lévy flight relation ($\gamma = 2/(\mu -1)$). Red circles represent experimental data for different school sizes

Furthermore, we tested the primary theoretical scaling relations for Lévy walks ($\gamma = 4 - \mu$) and Lévy flights ($\gamma = 2/(\mu - 1)$) to further cross-check the consistency of our model [33]. Figure 9 presents this comparison between our experimental $(\mu , \gamma )$ pairs and the idealized theoretical curves. While the experimental data do not perfectly overlap with the asymptotic predictions—a common feature in finite biological systems—they clearly follow the superdiffusive trend expected for the regime 2<μ<3.

The discrepancy between the theoretical predictions and experimental results can be attributed to the fact that scaling relations are typically derived for ideal, non-truncated Lévy processes in the asymptotic limit of infinite time. In our experimental setup, however, the step-length distribution is characterized by a truncated power law, and the movement is inherently bounded by the tank dimensions, which naturally induces deviations from idealized scaling.

**Fig. 10:**
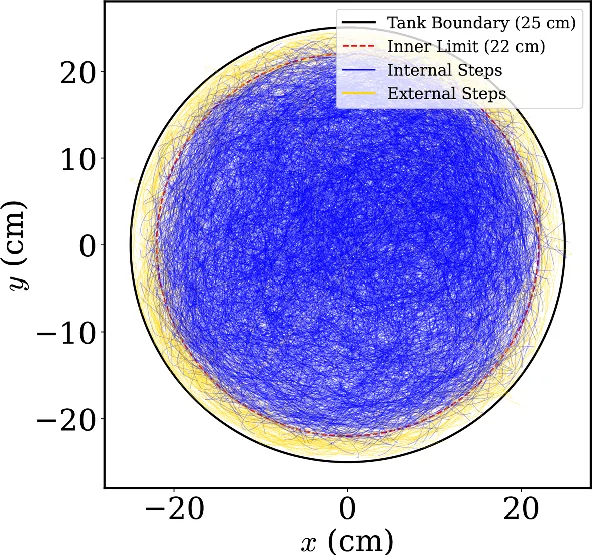
Spatial visualization of the experimental arena and detected movement steps. The black solid line represents the physical tank boundary (R=25 cm), while the red dashed line indicates the inner limit (R=22 cm) used for robustness analysis. Blue trajectories represent internal steps (more than 65% of displacement within the inner region), and gold trajectories indicate steps excluded due to edge effects

To verify the robustness of the reported transition from Lévy-like to Brownian dynamics and distinguish intrinsic collective effects from boundary-induced constraints, we performed a spatial filtering analysis restricted to the central region of the arena. We defined an exclusion zone of 3 cm adjacent to the physical boundary (25 cm) and analyzed only those steps for which trajectories occurred mostly (more than 65%) within the critical radius of 22 cm. Figure 10 illustrates this spatial classification, where internal steps (in blue) are distinguished from those potentially influenced by the boundary (in yellow). This filtering procedure helps isolate intrinsic social dynamics from geometric constraints, supporting the interpretation that the observed changes in stochastic patterns are driven primarily by inter-individual interactions rather than by the experimental boundary conditions.

**Fig. 11:**
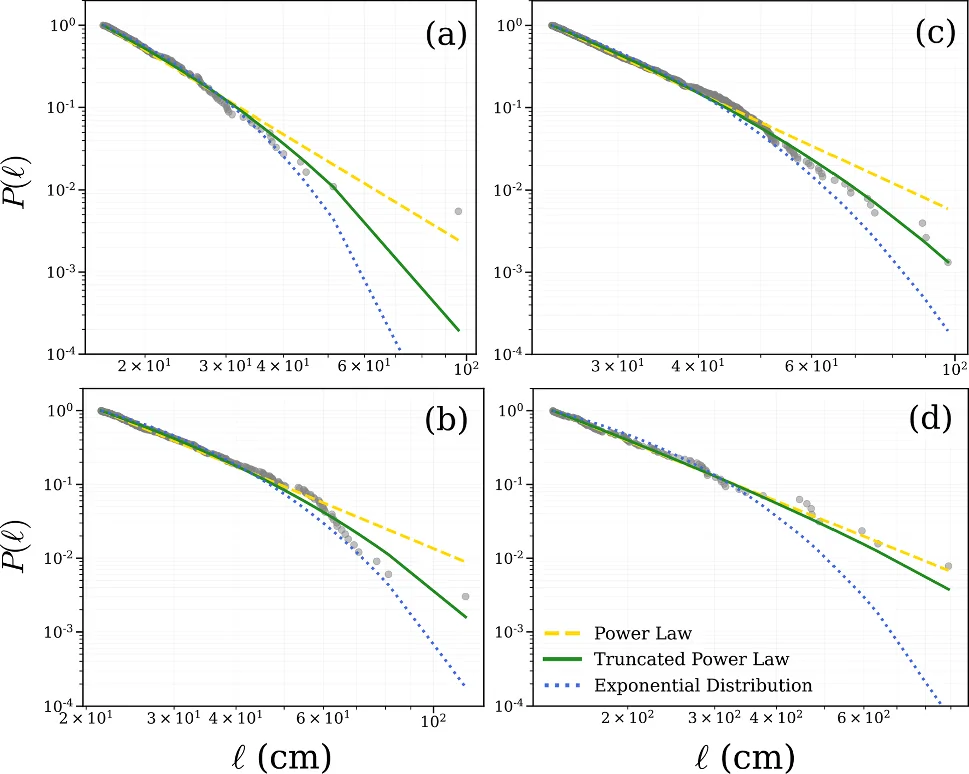
Cumulative step-length distributions considering only internal steps for different group sizes: **a** N=5, **b** N=10, **c** N=25, and **d** N=30. The solid and dashed lines represent the best fits for the truncated power law (green), pure power law (gold), and exponential (blue) models. The persistence of the transition in the central region confirms it as an intrinsic property of the collective system

The results obtained for the central region confirm the intrinsic nature of the dynamical transition. As shown in Fig. 11, even after removing edge effects, the heavy-tailed regime persists at low densities, with exponents $\mu = 2.33 \pm 0.34$ for N=5 and $\mu = 2.54 \pm 0.25$ for N=10, both situated within the characteristic range of Lévy walks (1<μ<3). For densities above the critical threshold (N≥15), the distributions consistently converge to the Brownian regime ($\mu \ge 3$), presenting $\mu = 3.0 \pm 0.3$ for N=25 and $\mu = 3.3 \pm 0.3$ for N=30.

This statistical consistency demonstrates that trajectory truncation and the consequent loss of anomalous diffusion are emergent properties of local density and the increased rate of neighbor shuffling. As the group becomes denser, the mean free path between inter-individual interactions becomes significantly smaller than the geometric scale of the arena. Therefore, the Brownian behavior observed in larger groups reflects a “social noise” regime that dominates individual dynamics even before physical confinement can act as the primary truncation mechanism.

The Lévy foraging hypothesis suggests that truncated Lévy walks could optimize random search processes in biological organisms [24, 25]. While our study does not directly test search efficiency, the emergence of Lévy-like patterns at low densities could be framed as a potential movement strategy that warrants further investigation in the context of collective exploration.

This discussion becomes more complex when we analyze the Lévy-walk dynamics in the movement of a fish about the center of mass of the school. In this case, (i) we are dealing with collective movement, where there are interactions between individuals, and (ii) the observed signature manifests itself in the rearrangement of the positions of the fish about the center of mass, that is, movements associated with the spatial reorganization of the school. Previous studies have already shown that schools of fish have a hierarchical structure, and these mechanisms influence several physical properties, such as polarization [16], avalanche dynamics [17], and influence networks [13]. The Lévy-walk pattern observed in $r_i$ may be another of these phenomena.

Work on the formation of pigeon flocks has revealed a relationship between the spatial position of individuals and their influential role in the group, allowing the construction of a directed hierarchical graph based on the observed interactions [13]. In schools of fish, a similar social structure is observed, with individuals in the leading positions exercising greater leadership and collective coordination [38, 39]. The immediate neighborhood also plays a central role in information propagation and behavioral synchronization [38].

Considering that the position of an individual in the school is often associated with its social position or function in the group, the presence of Lévy-walk characteristics in the positional rearrangements suggests that such movements are not random, but may reflect strategies for exploring new positions and efficiently exchanging functional roles within the group.

### *q*-Gaussian analysis

The relative position of zebrafish *i* with respect to the center of mass, $\textbf{r}_i(t)=\textbf{R}_i(t)-\textbf{R}_{CM}(t)$, is an important quantity for characterizing the internal dynamics of the group. Because the center of mass itself varies in time, this relative coordinate provides a dynamical description of the internal organization of the school. The associated fluctuations carry information about the temporal evolution of the group structure. Here, we quantify these fluctuations using the signed radial displacement increment

4 $$\begin{aligned}  &   \delta \xi _i(t)=|\textbf{r}_i(t+\delta _0)|-|\textbf{r}_i(t)|, \end{aligned}$$

where $\delta _0=1/30~\textrm{s}$ is the acquisition time interval, and $\delta \xi _i(t)$ represents the signed radial displacement increment of fish *i* relative to the school center of mass between consecutive frames.

**Fig. 12:**
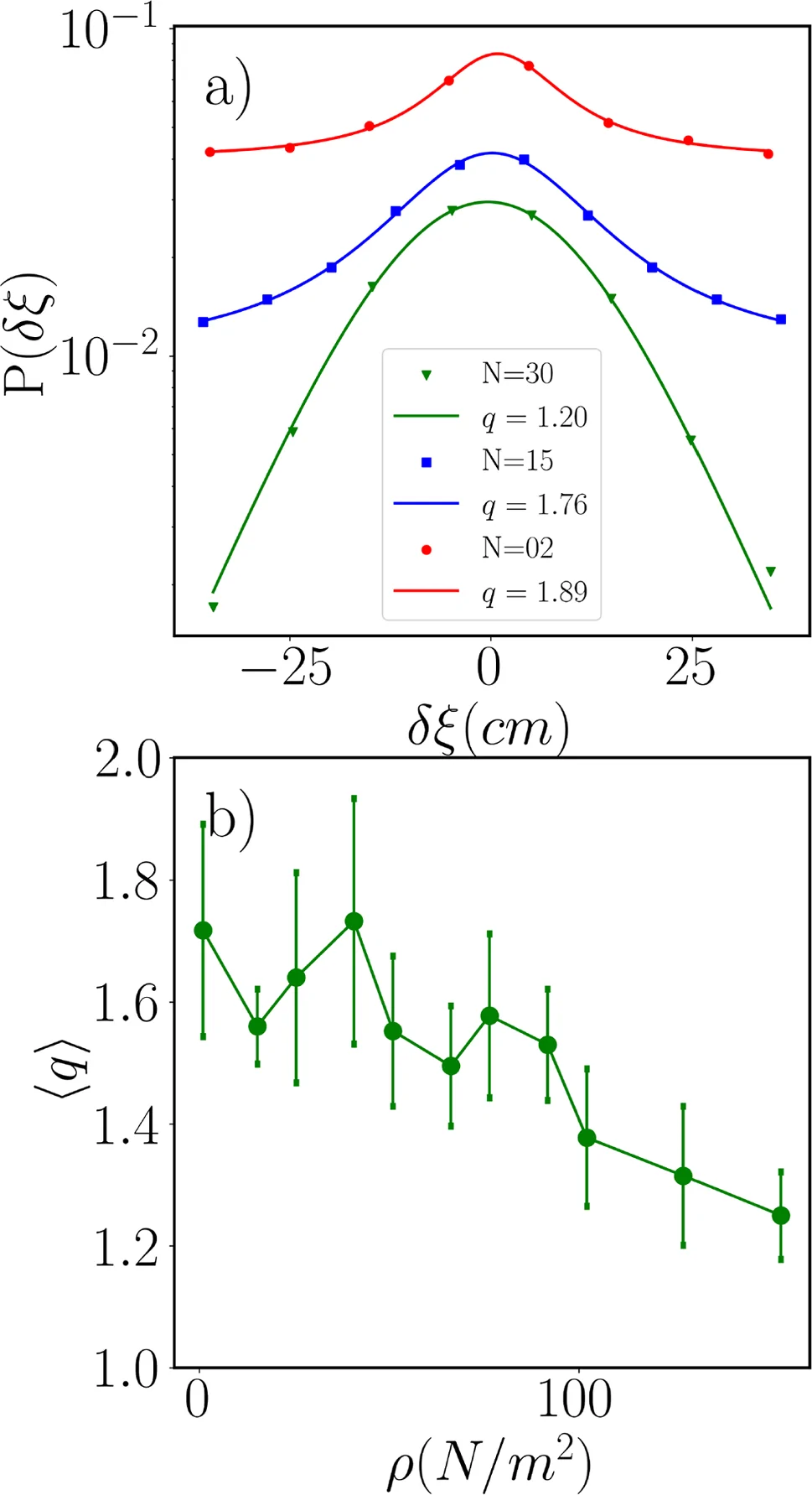
**a** Histograms of the signed radial displacement increments $\delta \xi$ of zebrafish relative to the center of mass of the group, with the corresponding *q*-Gaussian fits obtained from Eq. (5), for schools with N=2 (red circles), N=15 (blue squares), and N=30 (green triangles). **b** Fitted parameter *q* as a function of the fish density ρ

In Fig. 12a, we show the histograms of $\delta \xi$ for groups with N=2, N=15, and N=30 zebrafish. For $\rho >\rho _c$, the distribution tends toward a Gaussian-like shape, as illustrated by the curve for N=30. In this phase, the larger schools are organized into small domains, where correlations are stronger between fish within each domain but weaker between different domains. In contrast, for densities $\rho \le \rho _c$, a non-Gaussian distribution is observed, with a narrow peak close to zero and long tails. Since the analysis concerns signed fluctuations with respect to the center of mass, the resulting histograms are approximately symmetric about zero, reflecting the occurrence of positive and negative radial displacement increments. The fitted central value $\delta \xi _0$ remains close to zero.

A suitable function to describe the observed behavior is the *q*-Gaussian distribution, a generalization of the Gaussian function derived from Tsallis statistics [30], which is particularly effective in describing systems with non-Gaussian fluctuations, collective correlations, and heavy-tailed distributions [40]. The histograms in Fig. 12a can be fitted by the *q*-Gaussian probability density function

5 $$\begin{aligned}  &   P(\delta \xi ) = \frac{\sqrt{\beta _{0}}}{C_{q}} \left[ 1 + (q - 1) \beta _{0} (\delta \xi - \delta \xi _{0})^{2} \right] ^{\frac{1}{1 - q}}, \end{aligned}$$

where

6 $$\begin{aligned}  &   C_q=\sqrt{\pi }, \qquad q=1, \end{aligned}$$

and

7 $$\begin{aligned} C_q= \frac{ \sqrt{\pi }\, \Gamma \left( \frac{3-q}{2(q-1)}\right) }{ \sqrt{q-1}\, \Gamma \left( \frac{1}{q-1}\right) }, \qquad 1<q<3. \end{aligned}$$

Here, the constant $\beta _0$ determines the width of the distribution, while $\delta \xi _0$ represents its central value. In the limit q→1, the *q*-Gaussian recovers the standard Gaussian distribution. The parameter *q* therefore quantifies the deviation from Gaussian behavior: q=1 corresponds to ordinary Gaussian fluctuations, whereas q>1 indicates heavier tails and enhanced non-Gaussian fluctuations.

As shown in Fig. 12b, the fitted values of *q* decrease with increasing density. At low densities, the values lie in the range 1<q<2, indicating non-Gaussian, heavy-tailed fluctuations associated with collective correlations. As the density increases, *q* approaches unity, suggesting a progressive loss of correlations and a tendency toward Gaussian-like fluctuations.

**Fig. 13:**
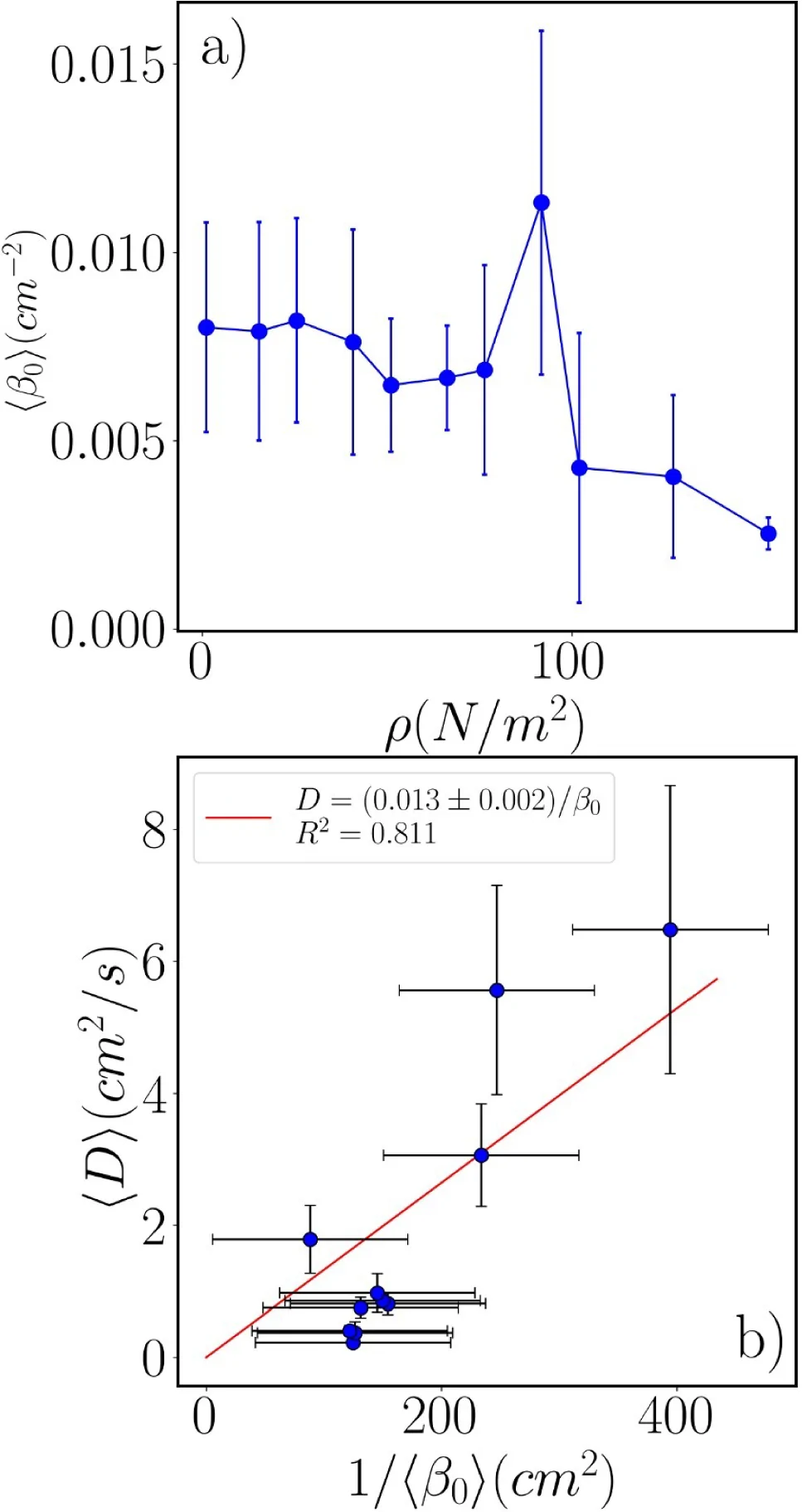
Analysis of the constant $\beta _0$. **a** Plot of $\beta _0$ versus density ρ. **b** Plot of the diffusion constant *D* versus $1/\beta _0$

**Fig. 15:**
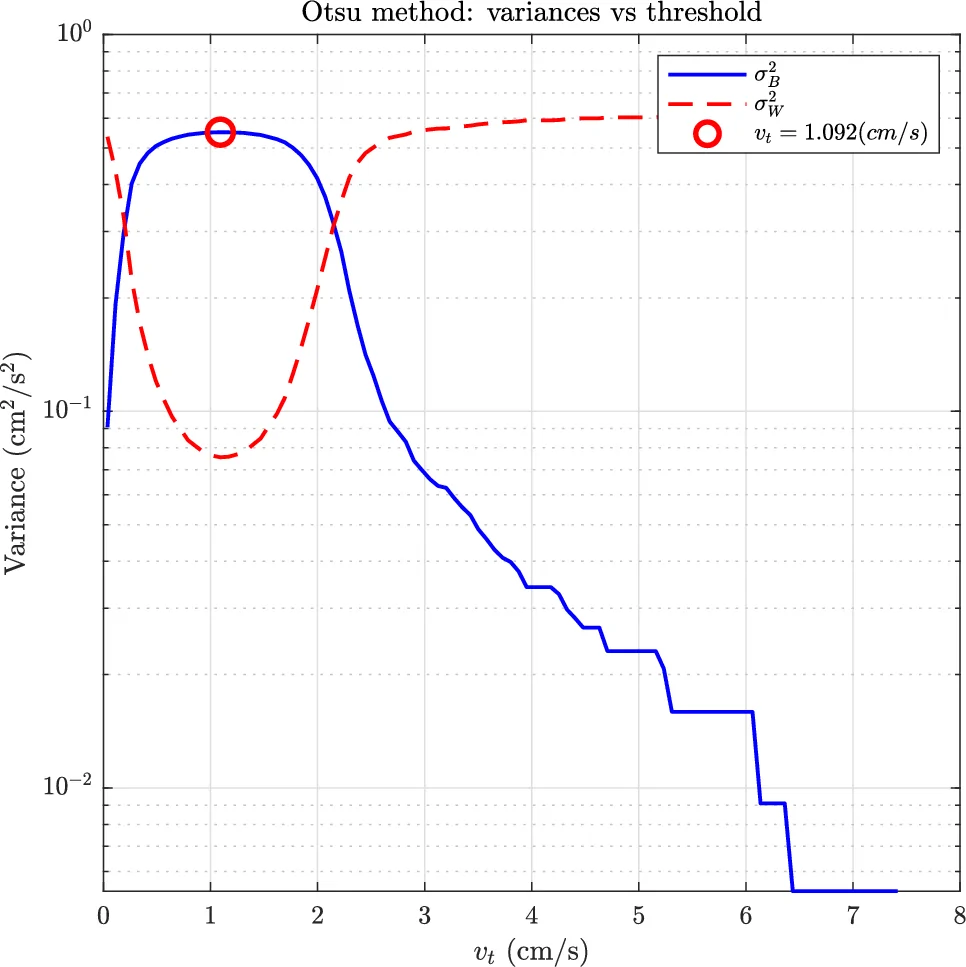
Within-class $\sigma _W^2$ (red dashed line) and between-class $\sigma _B^2$ (solid blue line) variances and a function of the threshold velocity $v_t$. The optimal value is indicated by the red circle, the maximum of $\sigma _B^2$ or minimum of $\sigma _W^2$. The Otsu method was applied to a 6.5 minutes time series of a single zebrafish recording experiment

The constant $\beta _0$ is associated with the inverse of an effective temperature, $T_{\textrm{eff}}$ [41], which measures the degree of fluctuation of the individual radial displacement increments around the center of mass in the generalized *q*-Gaussian distribution. Figure 13a shows how $\beta _0$ evolves as the school density increases. Considering $\beta _0\propto 1/T_{\textrm{eff}}$, the decrease in $\beta _0$ with increasing density indicates an increase in the effective temperature. This behavior is consistent with the analogy with a van der Waals gas, where the effective temperature increases with density.

In Fig. 13b, we present the diffusion coefficient *D* as a function of $1/\beta _0$, obtained from the data shown in Fig. 3 and Table 1. The fit performed using the relation $D=A/\beta _0$ shows a good correlation with the experimental results ($R^2=0.811$). The proportionality constant was found to be $A=0.013\pm 0.002~\textrm{s}^{-1}$. This result supports an analogy with the Stokes–Einstein relation, in which D∝T [42, 43]. The units are consistent with $[D]=\mathrm {cm^2/s}$ and $[1/\beta _0]=\mathrm {cm^2}$, resulting in $[A]=\textrm{s}^{-1}$.

## Summary and conclusions

We performed three empirical analyzes of the spatial self-organization of zebrafish within the school. First, we investigated the mean-square displacement of the fish position relative to the school’s center of mass. In low-density schools ($\rho \le \rho _{c}$), individual fish exhibit superdiffusive behavior, moving faster than Brownian walkers. At high densities ($\rho > \rho _{c}$), the behavior becomes close to Brownian and γ converging to 1.0, in agreement with the schooling-shoaling phase transition described in the literature [16, 17, 26].

Second, we then demonstrate that the dynamics of zebrafish relative to the school center of mass exhibit a Lévy-walk pattern. Our analysis reveals that this pattern, widely recognized for optimizing random searches in foraging contexts, also plays an essential role in the positional dynamics of fish within the school. This movement, characterized by alternating long and short jumps, can facilitate the efficient exploration of new positions and roles in the group. By investigating schools of different sizes, we identified that domains, especially under high-density conditions, directly influence the dynamics of changing positions and roles. We also observe the schooling-shoaling dynamic phase transition in the regime change from Lévy-walk dynamics to Brownian motion, as shown in Fig. 6.

Third, we performed an analysis based on the *q*-Gaussian distribution, applied to the signed radial displacement increments $\delta \xi$ relative to the school center of mass. The results indicate that the system progressively loses non-Gaussian correlations as the density of the shoal increases. In the same context, we observed behavior consistent with a Stokes–Einstein-like relation, in which the diffusion coefficient *D* is proportional to the effective temperature.

The study presented here highlights the complexity of the collective behavior of fish and its sensitivity to group density. The transition from a superdiffusive to a simple diffusion phase indicates that the increasing density significantly alters the interactions between individuals. This suggests that factors such as the size of the group, available space, and individual fish characteristics play a determining role in collective organization. The identification of regimes such as Lévy-walk dynamics indicates that fish movement is not random, but structured by information exchange within the group. Such findings reinforce the importance of experimental data and the application of quantitative tools in the study of biological systems.

## Figures and Tables

**Fig. 14:**
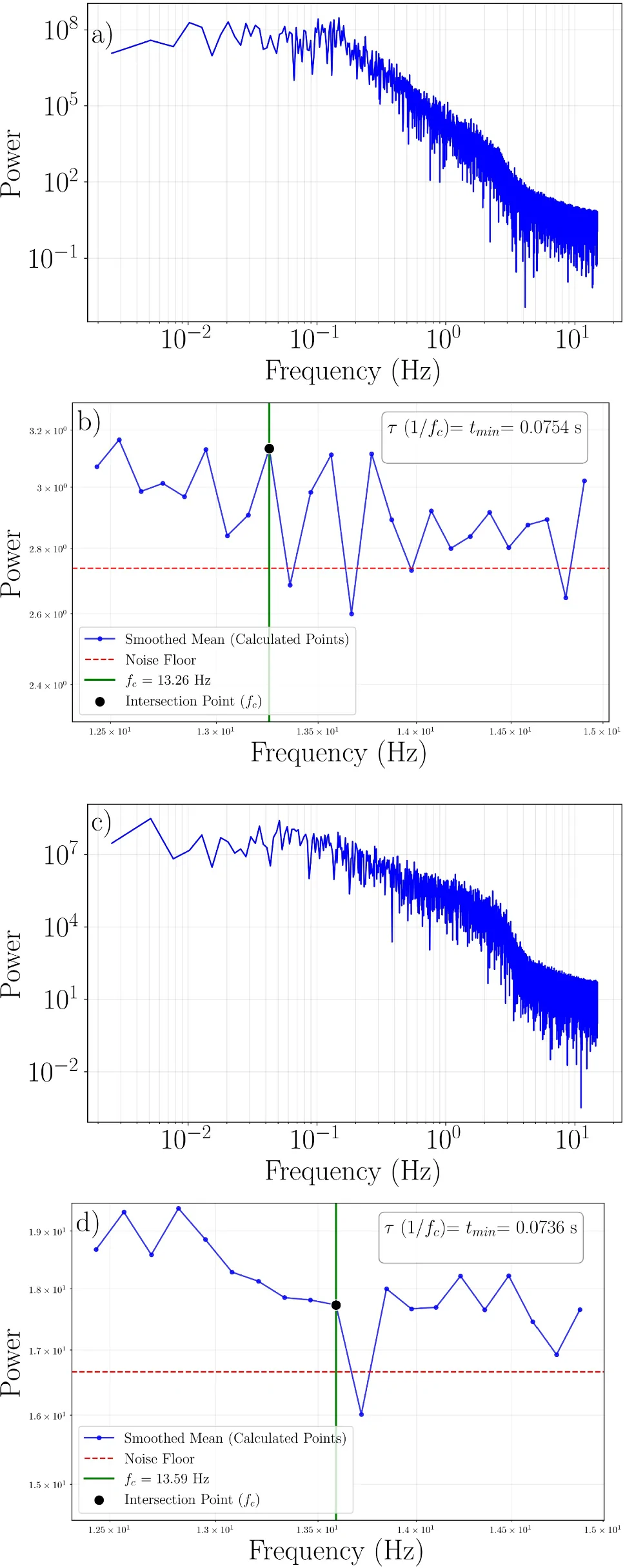
Power spectral density analysis. **a**, **c** Raw power spectra for N=8 and N=30, respectively. **b**, **d** Smoothed spectra with the red dashed line representing the noise floor and the green vertical line indicating the identified cutoff frequency $f_c$

**Fig. 16:**
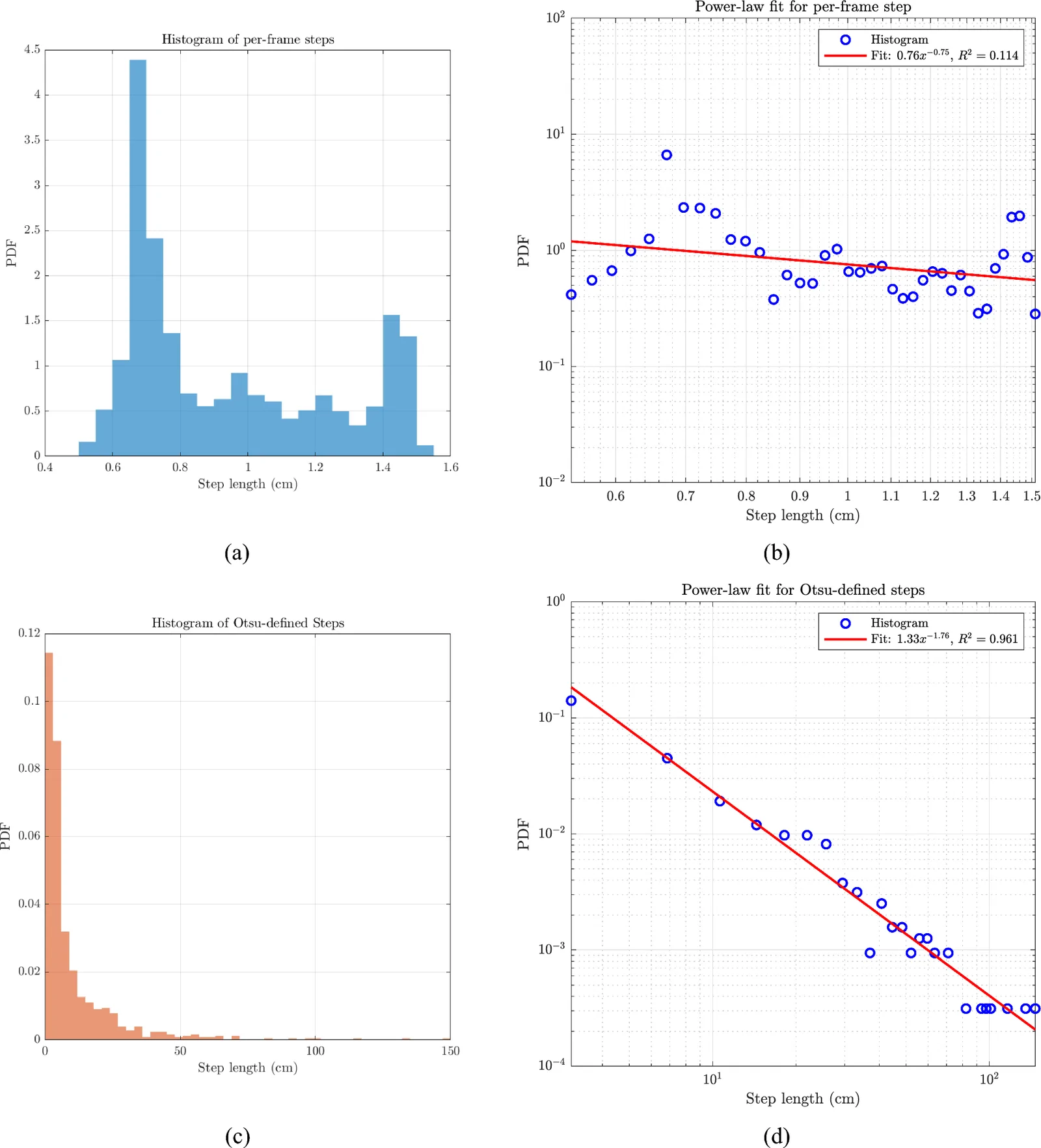
**a** Probability distribution function (PDF) of the fish step length measured at the acquisition frame rate (30 fps). **b** The same quantity computed using the Otsu method. The PDF obtained with fixed time windows exhibits multiple peaks and lacks symmetry, whereas the PDF obtained with Otsu-defined time windows follows a power-law behavior. **c** and **d** show the respective power-law fits, highlighting the difference in goodness of fit as indicated by the $R^2$ values

## Data Availability

Data will be made available on reasonable request.

## A: spectral convergence analysis for determining $t_{min}$

To ensure that the anomalous diffusion analysis is not influenced by instrumental noise, we implemented a methodology based on Power Spectral Density (PSD) convergence. This appendix details the steps taken to define the global lower bound ($t_{min}$) used in the mean-squared displacement (MSD) calculations.

### Methodology

The trajectories relative to the center of mass of zebrafish were analyzed in the frequency domain using a fast Fourier transform (FFT). The resulting power spectra exhibit a characteristic profile: a power-law decay at low frequencies (representing the biological signal) and a horizontal plateau at high frequencies (representing the white noise from the tracking system), see Fig. 14.

To objectively identify the transition between signal and noise, we applied a smoothing routine to the raw PSD data:

1. Smoothing: We calculated a moving average of the power spectrum using a window size of d=150 points in a range of m=1000 points near the Nyquist frequency (f≈15 Hz).
2. Background Noise Estimation: The instrumental background noise (noise floor) was defined as the average power of the higher-frequency components, where the signal is known to be dominated by tracking artifacts.
3. Convergence Criterion: We defined the cutoff frequency ($f_c$) as the first point of contact (zero crossing of the difference) between the smoothed signal (blue line) and the background noise (red dashed line), moving from low to high frequencies.
4. Time Conversion: The minimum time scale was then calculated as $t_{min} = 1/f_c$.

### Results

Figure 14a and c show the raw power spectra for group sizes of N=8 and N=30, respectively. Figure 14b and (d) show the corresponding smoothed curves and the identification of $f_c$.

As observed, the cutoff frequency remains remarkably stable across different group densities, with $f_c \approx 13.6$ Hz (corresponding to $t_{min} \approx 0.07$ s). This stability confirms that the high-frequency fluctuations are independent of the number of individuals and are purely a result of the experimental measurement limit. Consequently, a global $t_{min}$ of 0.07 s was adopted for all experimental conditions, providing a physically grounded and non-arbitrary window for the MSD power-law adjustments. Table 4 shows the values found in the spectral convergence analysis of all 55 measurements.

**Table 4:** Detailed parameters of the spectral convergence and MSD power-law fits for all 55 experimental realizations

Size	*D*	γ	$t_{min}$ (s)	$t_{max}$ (s)	$R^2$
N = 02	0.05	1.63	0.0753	1.333	0.996
	0.06	1.64	0.0732	1.333	0.997
	0.04	1.70	0.0722	1.333	0.997
	0.07	1.65	0.0732	1.333	0.994
	0.06	1.71	0.0709	1.333	0.997
N = 03	0.08	1.72	0.0716	1.333	0.997
	0.15	1.69	0.0706	1.333	0.996
	0.05	1.65	0.0796	2.000	0.997
	0.09	1.68	0.0788	2.000	0.998
	0.05	1.71	0.0716	1.333	0.998
N = 05	0.08	1.68	0.0804	1.333	0.998
	0.11	1.68	0.0772	1.667	0.996
	0.13	1.69	0.0804	1.667	0.997
	0.06	1.78	0.0772	1.667	0.997
	0.09	1.63	0.0772	1.667	0.995
N = 08	0.17	1.64	0.0804	1.333	0.996
	0.21	1.71	0.0804	2.000	0.996
	0.21	1.66	0.0772	2.000	0.996
	0.22	1.65	0.0717	1.500	0.996
	0.11	1.68	0.0772	2.000	0.997
N = 10	0.23	1.66	0.0772	2.000	0.996
	0.18	1.68	0.0804	1.667	0.996
	0.14	1.61	0.0736	1.667	0.996
	0.18	1.69	0.0788	2.000	0.996
	0.20	1.67	0.0772	2.000	0.999
N = 13	0.20	1.68	0.0765	1.667	0.996
	0.19	1.68	0.0709	1.667	0.997
	0.18	1.67	0.0804	1.667	0.997
	0.25	1.65	0.0722	1.667	0.995
	0.21	1.66	0.0729	1.667	0.996
N = 15	0.13	1.70	0.0736	2.167	0.997
	0.28	1.74	0.0722	2.000	0.998
	0.31	1.69	0.0804	2.667	0.997
	0.20	1.63	0.0703	2.167	0.996
	0.29	1.67	0.0796	2.667	0.998
N = 18	0.23	1.60	0.0703	1.667	0.998
	0.41	1.38	0.0716	1.667	0.996
	0.22	1.61	0.0709	2.333	0.997
	0.23	1.53	0.0729	2.000	0.998
	0.23	1.53	0.0684	2.000	0.998
N = 20	0.54	1.58	0.0729	2.000	0.998
	0.31	1.49	0.0716	2.667	0.997
	0.21	1.72	0.0796	2.000	0.997
	0.45	1.62	0.0788	2.000	0.999
	0.52	1.52	0.0724	1.667	0.997
N = 25	1.18	1.30	0.0796	2.000	0.998
	0.76	1.41	0.0709	1.667	0.998
	0.51	1.44	0.0709	1.667	0.997
	0.78	1.53	0.0709	2.000	0.998
	0.34	1.59	0.0709	2.000	0.997
N = 30	1.04	1.25	0.0736	2.000	0.996
	1.35	1.30	0.0716	1.667	0.997
	–	–	–	–	–
	–	–	–	–	–
	0.44	1.50	0.0772	1.667	0.997

## B: Adaptation of the Otsu method— threshold selection from unimodal velocity scale histograms

Consider the histogram of the magnitude of the speed of a particular fish in a school, denoted by *v*, shown in Fig. 5b. The speed of this fish varies in the interval $0< v < v_{max}$. We define $n_{j}$ as the number of events in which the speed of the fish is in the interval $[v_{j} - dv/2, v_{j} + dv/2]$, which are counted in the j-th bin of the histogram. The histogram is divided into *M* bins, each with width $dv = v_{max} / (M - 1)$. For simplicity, the histogram has been normalized and interpreted as a probability distribution,

8 $$\begin{aligned}  &   p_{j} = n_{j}/N,~~~~~~~ p_{j} > 0, ~ \sum _{j = 0}^{M} p_{j} = 1 \end{aligned}$$

We, then, divide the fish dynamics into two classes $C_{0}$ and $C_{1}$ (pausing and moving) by a threshold velocity $v_{t}$, in which $C_{0}$ denotes the fish with velocities $[0,..., v_{t}]$, and $C_{1}$ denotes the fish with velocities $[v_{t + 1},..., v_{M}]$.

The probabilities of occurrence of the classes and the average velocities of the classes, respectively, are given by:

9 $$\begin{aligned}  &   w_{0} = Pr(C_{0})= \sum _{j = 0}^{t} p_{j}= w(v_{t}) \end{aligned}$$

10 $$\begin{aligned}  &   w_{1} = Pr(C_{1})= \sum _{j = t + 1}^{M} p_{j} = 1 - w(v_{t}) \end{aligned}$$

and

11 $$\begin{aligned}  &   \eta _{0} = \sum _{j = 0}^{t} v_{j} Pr (v_j|C_{0}) = \sum _{j = 0}^{t} \frac{v_{j} p_{j}}{w_{0}} = \eta (t)/w(v_{t}) \end{aligned}$$

12 $$\begin{aligned}  &   \eta _{1} = \sum _{j = t + 1}^{M} v_{j} Pr (v_j|C_{1}) = \sum _{j = t + 1}^{M} \frac{v_{j} p_{j}}{w_{1}} = \frac{(\eta _{T} - \eta (t))}{(1 - w(v_{t}))}\nonumber \\ \end{aligned}$$

where

13 $$\begin{aligned}  &   \eta _{T} = \eta (v_{M}) = \sum _{j = 1}^{M} v_{j} p_{j} \end{aligned}$$

is the average total speed of the fish, in the limit $M \rightarrow \infty$. The following relation can be readily verified for any value of t:

14 $$\begin{aligned}  &   w_{0}\eta _{0} + w_{1}\eta _{1} = \eta _{T}. \end{aligned}$$

The class variances are given by:

15 $$\begin{aligned}  &   \sigma _{0}^2 = \sum _{j = 1}^{t} (v_{j} - \eta _{0})^2 Pr(v_j|C_{0}) = \sum _{j = 1}^{t} (v_{j} - \eta _{0})^2 p_{j}/w_{0} \end{aligned}$$

16 $$\begin{aligned}  &   \sigma _{1}^2 = \sum _{j = t + 1}^{M} (v_{j} - \eta _{1})^2 Pr(v_j|C_{1}) = \sum _{j = t + 1}^{M} (v_{j} - \eta _{1})^2 p_{j}/w_{1}.\nonumber \\ \end{aligned}$$

These require second-order cumulative moments.

To assess the “goodness” of the threshold velocity ($v_{t}$), we will introduce the following discriminant criteria measures (or class separability measures) used in discriminant analysis:

The within-class variance is:

17 $$\begin{aligned}  &   \sigma _{W}^2 = w_{0} \sigma _{0}^2 + w_{1} \sigma _{1}^2. \end{aligned}$$

The between-class variance is:

18 $$\begin{aligned} \sigma _{B}^2&= w_{0} (\eta _{0} - \eta _{T})^2 + w_{1} (\eta _{1} - \eta _{T})^2 \nonumber \\ \quad&= w_{0} w_{1}(\eta _{1} - \eta _{0})^2. \end{aligned}$$

The total variance is:

19 $$\begin{aligned}  &   \sigma _{T}^2 = \sum _{j = 1}^{M} (v_j - \eta _{T})^2 p_{j} \end{aligned}$$

where

20 $$\begin{aligned}  &   \sigma _{T}^2 = \sigma _{W}^2 + \sigma _{B}^2 \end{aligned}$$

Otsu’s adapted method determines the optimal threshold $v_{t}$ by maximizing the between-class variance ($\sigma _{B}$), or equivalently minimizing the within-class variance ($\sigma _{W}$). This is because the total variance, which is the sum of the between- and within-class variance, remains constant for any partition.

In Fig. 15, we show the values of $\sigma _{B}^2$ and $\sigma _W^2$ and a function of the $v_t$. Although $v_t$ can assume any values between 0 and $v_M$, it is discretized into *M* bins. The discretization removes the complexity to find the maximum in a widely distributed dataset and turns the problem into a simple task to find the maximum in a vector with M elements. In our analysis, *M* is approximately 100.

The difference between before and after the Otsu method can be seen in Fig. 16.

21 $$\begin{aligned}  &   v_{t} = arg\left\{ \underset{0 \le t \le M}{max}\ \left\{ \sigma _{B}^2 \right\} \right\} = arg\left\{ \underset{0 \le t \le M}{min}\ \left\{ \sigma _{W}^2 \right\} \right\} \nonumber \\ \end{aligned}$$
